# Supplementary figures and images for: Engineered sex ratio distortion by X-shredding in the global agricultural pest Ceratitis capitata
Source: BMC Biol. 2021 Apr 16;19:78. doi: 10.1186/s12915-021-01010-7 (PMC8051031; doi:10.1186/s12915-021-01010-7)

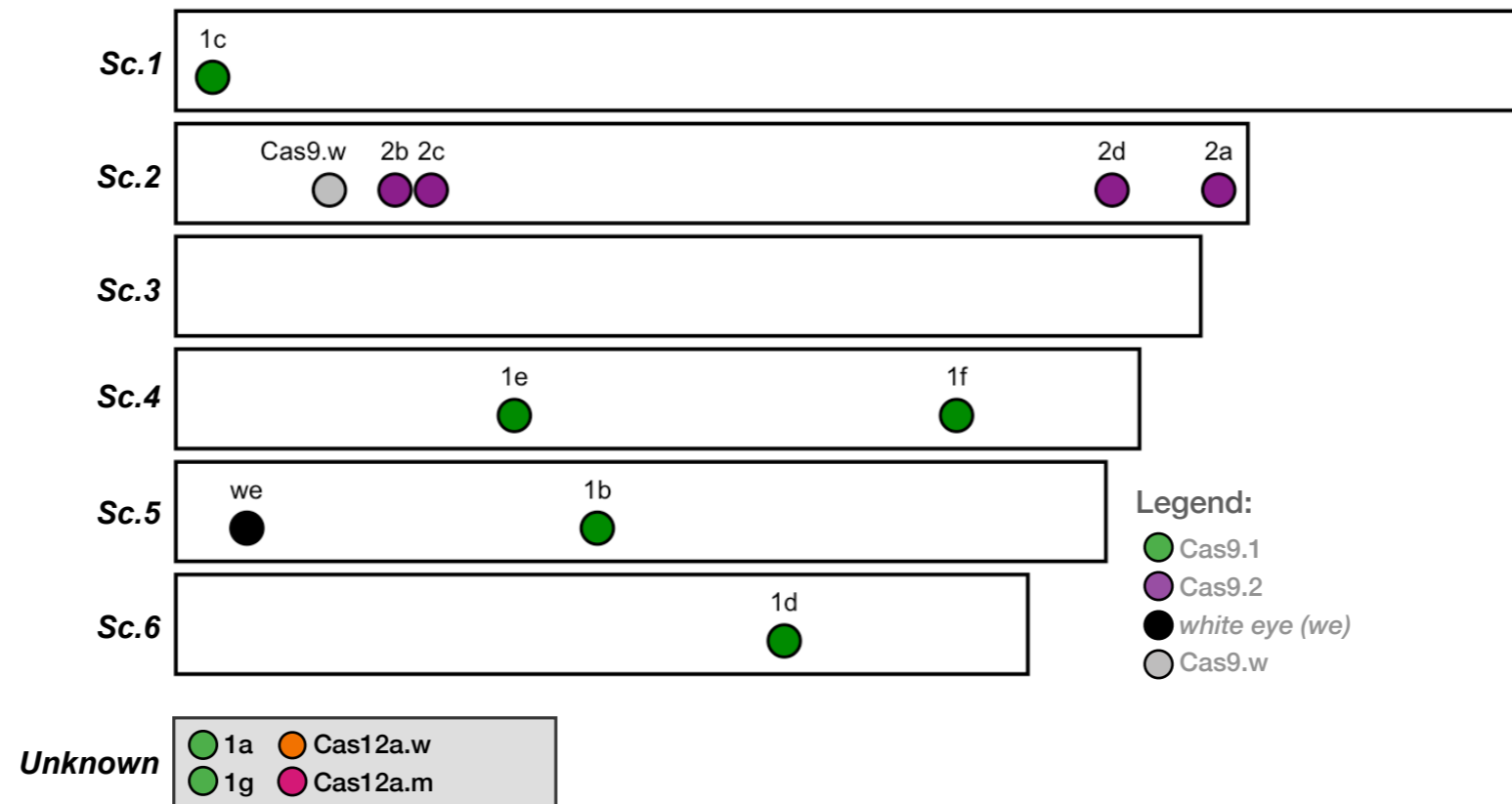

Supplement: Supplementary file 2 — Additional file 2: Figure S1. Predicted transgene integration sites within the EgII_Ccap3.2.1 assembly. [file 12915_2021_1010_MOESM2_ESM.pdf]

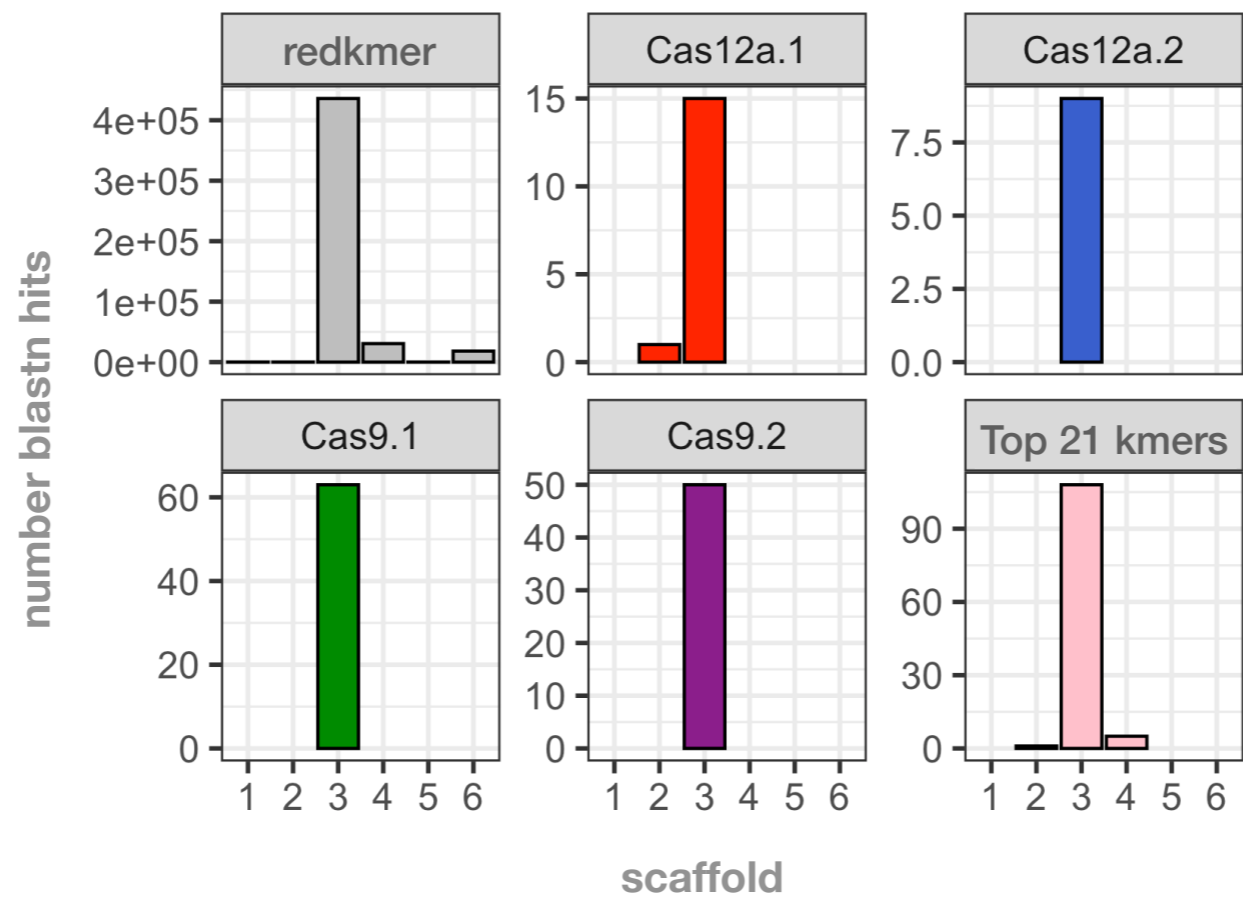

Supplement: Supplementary file 5 — Additional file 5: Figure S2. BLASTN hits of selected kmers to the Ccap3.2 assembly. [file 12915_2021_1010_MOESM5_ESM.pdf]

**A**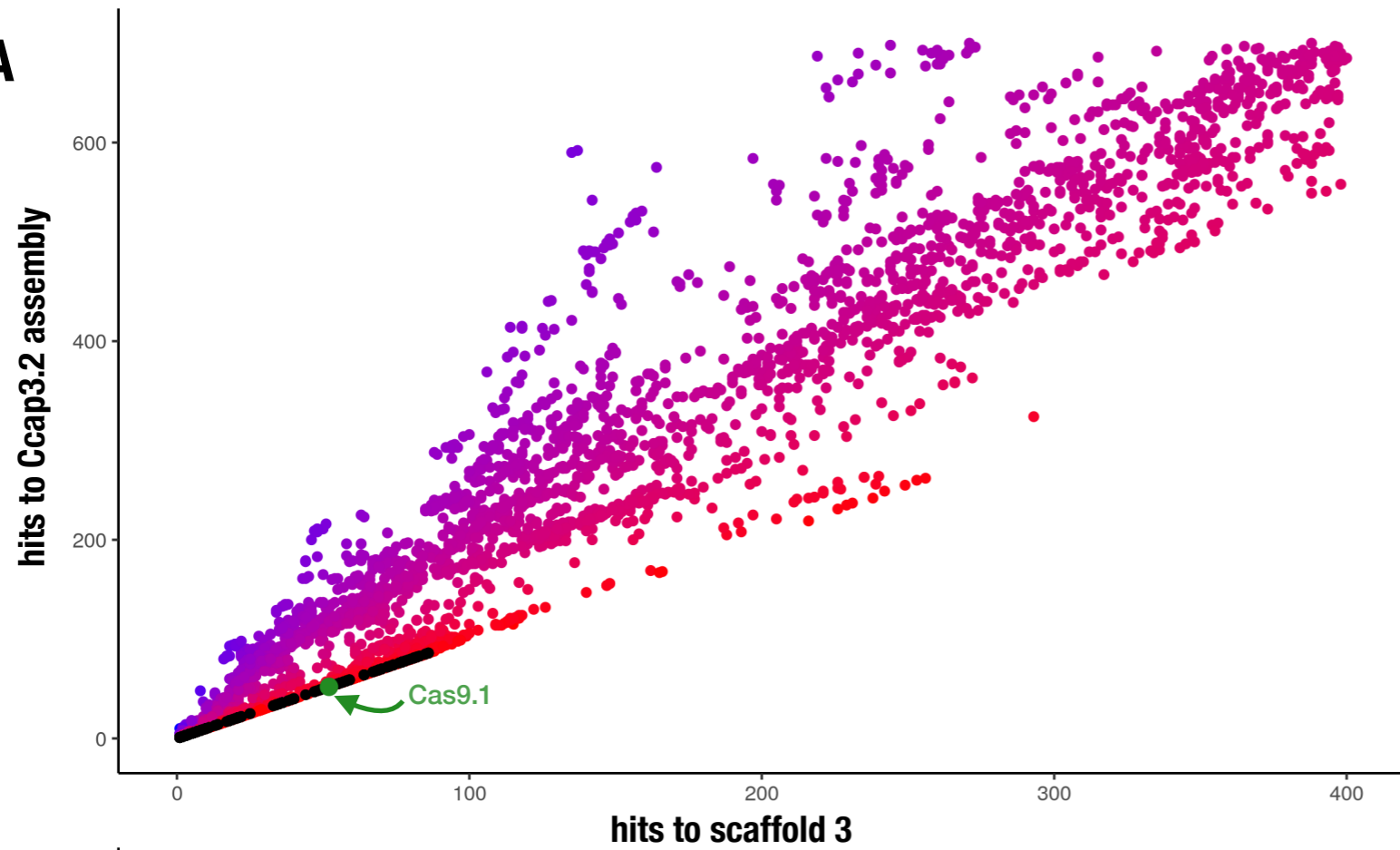**B**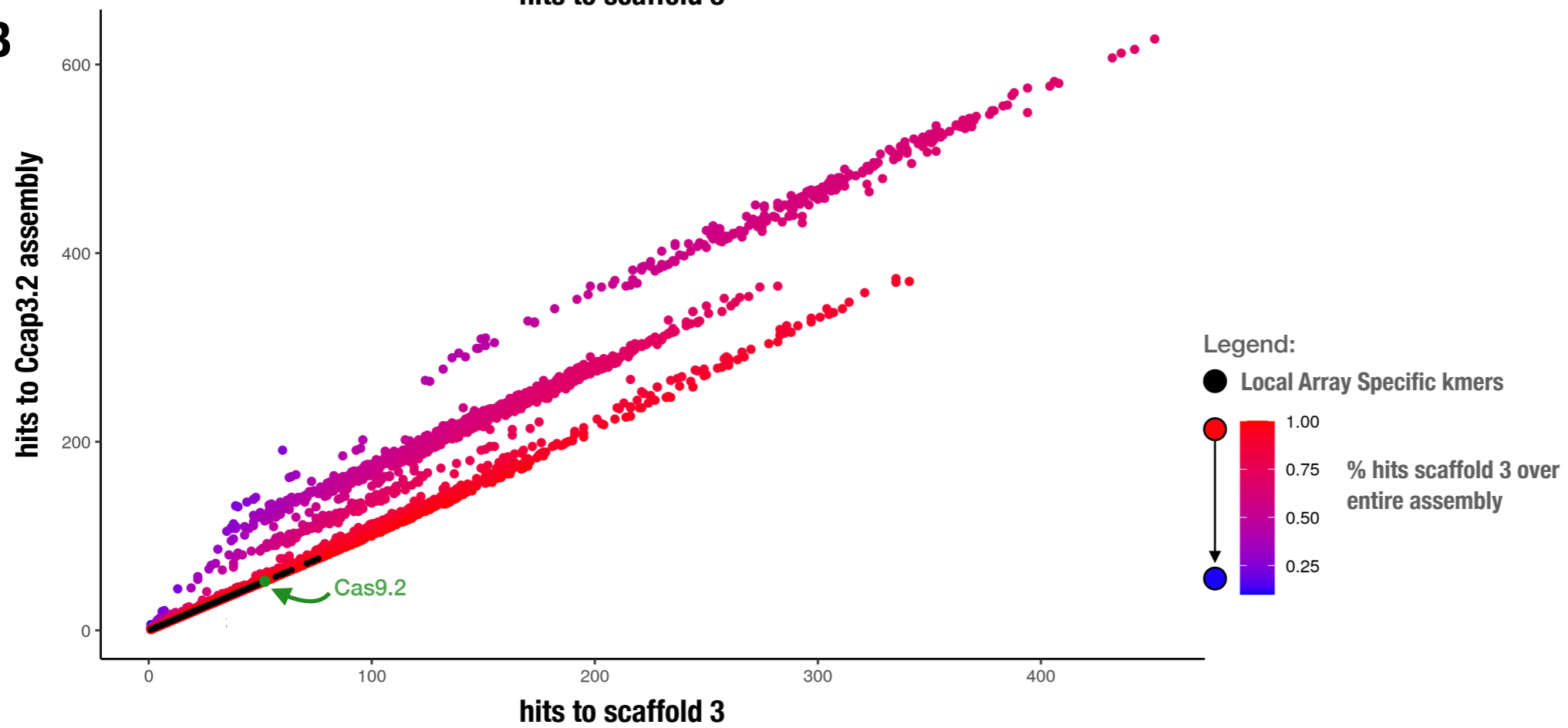

Supplement: Supplementary file 7 — Additional file 7: Figure S3. Specificity and abundance of kmers originating in the target regions. [file 12915_2021_1010_MOESM7_ESM.pdf]
